# Editorial: Update on eosinophil-associated diseases

**DOI:** 10.3389/falgy.2025.1740057

**Published:** 2025-12-03

**Authors:** Nicola Laura Diny, Yoshiyuki Yamada, Nives Zimmermann

**Affiliations:** 1 Institute of Clinical Chemistry and Clinical Pharmacology, Universitätsklinikum Bonn, Bonn, Germany; 2 Department of Pediatrics, Tokai University School of Medicine, Kanagawa, Japan; 3 Departments of Pathology and Laboratory Medicine, and Pediatrics, University of Cincinnati College of Medicine, Cincinnati, OH, United States

**Keywords:** eosinophil-associated disorders, eosinophilic gastrointestinal disorders, hypereosinophilic syndrome, eosinophilic granulomatosis with polyangiitis, allergic asthma, eosinophilic esophagitis, heart disease, inflammatory bowel diseases

Eosinophil-associated disorders (EADs), including eosinophilic gastrointestinal disorders, hypereosinophilic syndrome, and eosinophilic granulomatosis with polyangiitis are a heterogeneous group of conditions characterized by blood and/or tissue hypereosinophilia leading to eosinophil-mediated clinical manifestations. Recently, biologic therapies that target eosinophils have been developed and approved for some of the EADs, promising improved treatment options and outcomes for patients with these rare diseases. However, responsiveness to eosinophil-targeted therapies is not universal in all EADs and predicting which patients will respond to which biologic therapy are some of the unanswered questions, which necessitate both basic and clinical studies into the mechanisms of EADs. This Research Topic gathers different contributions on different eosinophil-associated diseases, including allergic asthma, eosinophilic esophagitis, heart disease and inflammatory bowel diseases. Through clinical, translational and basic science studies and reviews the role of eosinophil is investigated across this range of diseases.

## Cardiac eosinophils: from infiltration to immunoregulation

Hypereosinophilia is frequently associated with cardiac complications (1). The work presented here by Sunusi et al., builds on and confirms previous findings on the role of eosinophils in autoimmune-mediated and hypereosinophilia-induced heart disease (2–4). Induction of experimental autoimmune myocarditis in a mouse model leads to eosinophil infiltration in the heart, and is associated with changes in eosinophil gene expression and phenotype. The authors find that cardiac eosinophils upregulate genes associated with immune response, cell migration, antigen processing and presentation, as well as pattern recognition receptors during autoimmune inflammation. Eosinophils in autoimmune myocarditis also demonstrated increased expression of the regulatory molecule CD274, at both mRNA and protein level. Interestingly, CD274 was upregulated on eosinophils *in vitro* after stimulation with IFN*γ* or LPS, both of which are associated with this autoimmune disease model, reflecting the *in vivo* phenotype.

## Eosinophils in the intestinal immune system and inflammatory bowel disease (IBD)

Eosinophils are increasingly recognized as key players in the intestinal immune system, contributing to the antimicrobial response and immune homeostasis (5–8). Their role in inflammatory bowel diseases however, remains controversial. On one hand, increased eosinophil numbers and evidence of degranulation in IBD point toward a pathologic role (9). On the other hand, evidence from animal models suggests that eosinophils may play immunoregulatory roles, dampening inflammation (10, 11). The review here by Tomii and Kano highlights relevant articles on both sides, revealing a complex and likely context-dependent role for eosinophils. They further postulate the hypothesis that eosinophil-derived reactive oxygen species (ROS), particularly hydrogen peroxide, contribute to a regulatory function in maintaining intestinal barrier integrity. In this model, appropriate levels of ROS from eosinophils and other cell types promote epithelial growth, mucus secretion, and pathogen control. Excess ROS, however, which may be released during uncontrolled eosinophil activation, causes tissue damage and amplifies inflammation. The authors further propose a mechanism by which the inhibitory receptor Siglec-8 may be blocked by diet-derived ligands. The relevance of these pathways for IBD pathogenesis remains to be tested experimentally.

## Respiratory tract eosinophils and heterogeneity

Similarly, eosinophils have been shown to have both host-protective and host-destructive roles in the respiratory tract. Sasaki et al. summarize what is known about the asthma-viral infection crosstalk of eosinophils. Briefly, in type 2 cytokine mediated airway inflammation such as asthma, eosinophils contribute to tissue injury (12–15). In contrast, when activated by interferons such as in viral infections, eosinophils have antiviral actions and contribute to host protection (16, 17). However, eosinophils in type 2 cytokine environments have impaired antiviral activity and patients with asthma are vulnerable to viral infections (18–21) suggesting impaired eosinophil antiviral function is involved in asthma exacerbation.

Another mechanism for host protective vs. destructive roles of eosinophils is in the heterogeneity of eosinophils, with different subtypes and/or activation states leading to different outcomes (11, 22, 23). Graf et al. provide a review of eosinophil recruitment and heterogeneity during allergic airway inflammation. Particularly, in the lungs, a subset of resident eosinophils have immune regulatory and homeostatic functions. In contrast, inflammatory eosinophils which expand in allergic airway inflammation in mouse models and patients with asthma have a pro-inflammatory phenotype and function. Thus, subtypes or activation states of eosinophils may guide their homeostatic or pro-inflammatory role, and the mechanisms are being actively elucidated.

## Eosinophilic esophagitis and emerging concepts

Eosinophilic esophagitis (EoE) is a chronic, immune-mediated disease characterized by marked eosinophil infiltration of the esophageal epithelium, leading to symptoms such as dysphagia, vomiting, and feeding difficulties. The prevalence of EoE has risen rapidly in Western countries over the past two decades, while it remains relatively rare in Asia (24). Recent contributions have expanded the scope of knowledge from clinical observation to the discovery of biomarkers and environmental interactions.

Noble et al. conducted a PRISMA-based systematic review of 37 studies (2017–2024) investigating over 80 non-invasive biomarkers for EoE, across blood, saliva, breath and urine, as well as minimally-invasive esophageal sampling techniques such as Cytosponge, EST, and brushing. While peripheral eosinophil counts have been the focus of the majority of studies, their diagnostic reliability remains limited. In contrast, eosinophil-derived proteins (EDN, MBP-1, ECP) and cytokines such as eotaxin-3 and TGF-*β* emerged as promising indicators. Esophageal-specific sampling correlated best with histologic activity, underscoring the feasibility of biopsy-sparing diagnostics.

Sekar et al. examined the impact of aeroallergens—including pollen, mites, and molds—on EoE pathogenesis. They highlight that inhaled antigens, through epithelial barrier disruption, can activate Th2 cytokines (IL-4, IL-5, IL-13) and amplify inflammation, revealing parallels between respiratory and gastrointestinal allergic mechanisms. Seasonal variation and atopic comorbidity support a link between respiratory sensitization and EoE exacerbation. The review also notes that subcutaneous immunotherapy may improve EoE outcomes, whereas sublingual immunotherapy can occasionally induce EoE, emphasizing the need for careful therapeutic balance.

Dominicus et al. present a retrospective analysis of 79 children diagnosed with EoE between 2014 and 2020. Their study revealed that 14 patients had normal appearing mucosa yet histologic evidence of EoE, emphasizing the need for additional diagnostic evaluation even in the absence of grossly visible changes. Treatment primarily included proton pump inhibitors, with adjunctive swallowed budesonide or elimination diets achieving remission in approximately 87% of cases, though relapses were common. The findings highlight the necessity for long-term monitoring and individualized management strategies.

Collectively, these EoE studies shift the disease concept from a purely food-driven reaction toward a systemic, type 2 inflammation–dominant disorder influenced by genetic, epithelial, and environmental factors. The prospective integration of non-invasive biomarker assessment, environmental control, and targeted biologics holds the potential to transform EoE care into a precision medicine paradigm.

## Perspective and future directions

Across organ systems, the studies in this Research Topic reveal the dual and context-dependent nature of eosinophils—from tissue repair and immune regulation to fibrosis and chronic inflammation. Functional heterogeneity, tissue adaptation, and the balance between activation and regulation emerge as recurring themes. Biologic therapies targeting IL-5, IL-4R*α*, or Siglec-8 underscore the clinical potential of modulating eosinophil pathways but also demand robust biomarkers to predict response. Integration of omics-based profiling, imaging, and minimally invasive monitoring will be crucial to track eosinophil dynamics longitudinally. Ultimately, the collective efforts reflected here pave the way toward a mechanistic and personalized approach to eosinophil-associated diseases.
