# Correction: Editorial: Update on eosinophil-associated diseases

**DOI:** 10.3389/falgy.2025.1762252

**Published:** 2025-12-18

**Authors:** Nicola Laura Diny, Yoshiyuki Yamada, Nives Zimmermann

**Affiliations:** 1 Institute of Clinical Chemistry and Clinical Pharmacology, Universitätsklinikum Bonn, Bonn, Germany; 2 Department of Pediatrics, Tokai University School of Medicine, Kanagawa, Japan; 3 Departments of Pathology and Laboratory Medicine, and Pediatrics, University of Cincinnati College of Medicine, Cincinnati, OH, United States

**Keywords:** eosinophil-associated disorders, eosinophilic gastrointestinal disorders, hypereosinophilic syndrome, eosinophilic granulomatosis with polyangiitis, allergic asthma, eosinophilic esophagitis, heart disease, inflammatory bowel diseases

The authors were erroneously assigned to all affiliations. This previously appeared as: “Nicola Laura Diny 1,2,3, Yoshiyuki Yamada 1,2,3 and Nives Zimmermann 1,2,3*”

Instead, authors should be assigned to affiliations 1, 2, and 3 respectively. This should appear as: “Nicola Laura Diny 1, Yoshiyuki Yamada 2 and Nives Zimmermann 3*”.

The original version of this article has been updated.

